# Evaluating the Risk of Postoperative Infection and Complications in Lumbar Spine Surgery Patients with Preoperative Methicillin-resistant *Staphylococcus aureus* (MRSA) Colonization

**DOI:** 10.1007/s00402-025-06036-y

**Published:** 2025-08-19

**Authors:** Sri Tummala, Drew Haslam, David Gibbs, Jason Alder, Joseph Chavarria, Ioannis Avramis, James Rizkalla

**Affiliations:** 1 Department of Orthopaedic Surgery, Baylor University Medical Center, Dallas, USA; 2 Texas A&M College of Medicine, Dallas, USA

**Keywords:** Methicillin-resistant *Staphylococcus aureus*, *S. aureus*, Lumbar spine surgery, Surgical site infection, Complications, Outcomes

## Abstract

**Introduction:**

Preoperative methicillin-resistant *Staphylococcus aureus* (MRSA) colonization is a known risk factor for surgical site infections (SSIs) in orthopaedic procedures. However, its impact on a comprehensive range of postoperative complications, particularly in elective lumbar spine surgery (LSS), remains unexplored. This study evaluated the association between preoperative MRSA colonization and a comprehensive set of 30-day postoperative outcomes in patients undergoing LSS.

**Materials and methods:**

A retrospective cohort study was conducted using the TriNetX multi-institutional database, including 440,336 patients undergoing elective LSS. Patients were stratified into MRSA-colonized (*n* = 3,711; 0.84%) and non-colonized controls (*n* = 436,625). Propensity score matching (1:1) adjusted for demographics and comorbidities (age, race, sex, obesity, diabetes, tobacco use, malnutrition, chronic kidney disease), yielding balanced cohorts of 3,706 patients each. Primary outcomes included 30-day mortality, SSIs, systemic infections (sepsis, pneumonia), hematologic complications (anemia, transfusions), renal failure, and thromboembolic events. Risk ratios (RR) with 95% confidence intervals were calculated.

**Results:**

MRSA-colonized patients exhibited significantly higher complication risks versus matched controls: Wound complications: superficial SSI (RR = 2.291, *p* < 0.01), deep SSI (RR = 2.566, *p* < 0.01), wound dehiscence (RR = 1.722, *p* < 0.01). Systemic Infections: sepsis (RR = 2.865, *p* < 0.001), pneumonia (RR = 2.212, *p* < 0.001). Hematologic/renal events: transfusion (RR = 2.382, *p* < 0.001), anemia (RR = 2.826, *p* < 0.001), acute kidney failure (RR = 2.344, *p* < 0.001). Mortality: all-cause mortality was 2.05-fold higher (RR = 2.046, *p* < 0.01). Demographic analysis identified five major risk factors: obesity, diabetes, tobacco use, malnutrition, and chronic kidney disease (CKD) as independent predictors of MRSA colonization.

**Conclusions:**

Preoperative MRSA colonization is independently associated with significantly elevated risks of mortality, wound complications, systemic infections, hematologic morbidity, and acute renal injury after elective LSS. Clinically, preoperative recognition of MRSA colonization could prompt implementation of multimodal decolonization protocols and targeted counseling regarding heightened complication risks. This risk-stratified approach may optimize perioperative management and improve outcomes in high-risk LSS patients.

**Supplementary Information:**

The online version contains supplementary material available at 10.1007/s00402-025-06036-y.

## Introduction

Postoperative infections following elective spinal surgery pose substantial challenges in clinical outcomes and healthcare resource management [1, 2]. Annually, over 650,000 spinal surgeries are performed in the United States, with postoperative infection rates for instrumented procedures historically ranging between 0.2% and 4.7%, averaging approximately 2% [1–6]. Predominantly, these infections result from Gram-positive bacteria, typically originating from the patient’s skin flora, with *Staphylococcus aureus* (*S. aureus*) and *Staphylococcus epidermidis* being the most common pathogens [7]. Although *methicillin-sensitive Staphylococcus aureus* (MSSA) continues to be prevalent, there has been a concerning increase in the incidence of infections caused by *methicillin-resistant S. aureus* (MRSA) [7]. Colonization of the nasal passages with MRSA notably increases the risk for subsequent MRSA infections and associated bacteremia, emphasizing the critical need for effective preoperative detection and management strategies [8–10].

Within orthopaedic surgical settings, it is well established that colonization by *S. aureus* is known to significantly heighten the risk of surgical site infections (SSI) and periprosthetic joint infections [11–13]. Approximately one-third of the general population are carriers of *S. aureus*, predominantly in the nasal passages [14], and MRSA specifically has been implicated in 23% of primary total hip arthroplasty (THA) infections and 21% of revision THA infections, underscoring its prevalence and significant role in adverse postoperative outcomes in musculoskeletal contexts [15]. Managing MRSA infections clinically is notably challenging, with such infections associated with elevated mortality risks and extended hospitalizations compared to MSSA infections [15]. Specifically, the mortality associated with MRSA infections is reportedly twice that of MSSA infections, highlighting the critical importance of preoperative MRSA management strategies [16]. Therefore, comprehensive insights into these risks are essential for optimizing patient counseling, perioperative planning, and targeted interventions aimed at managing postoperative complications [15].

Medical organizations including the International Consensus Meeting on Musculoskeletal Infection (ICM), the European Bone and Joint Infection Society (EBJIS), and the Musculoskeletal Infection Society (MSIS) currently lack definitive recommendations for preoperative MRSA screening and decolonization due to inconsistent evidence [17–19]. Although previous studies consistently demonstrate increased invasive MRSA infection risk among colonized individuals in various total joint procedures [20–23], population-based research directly examining the link between MRSA colonization and an extensive variety of postoperative complications in elective LSS patients remains limited.

To our knowledge, this study represents the first comprehensive analysis utilizing a multi-institutional database to evaluate patient demographics, clinical characteristics, and postoperative complication rates in LSS patients with documented preoperative MRSA colonization. Findings from this investigation aim to inform the establishment of evidence-based preoperative management protocols and targeted patient counseling, thereby optimizing clinical outcomes following LSS. The authors hypothesized that MRSA-colonized LSS patients will experience significantly higher rates of postoperative complications compared to non-colonized patients.

## Methods

### Data acquisition

This retrospective study leveraged the TriNetX Research database (https://trinetx.com, Cambridge, MA, USA). TriNetX aggregates electronic health record data encompassing clinical diagnoses, medical procedures, medication histories, laboratory test results, and genetic data from approximately 150 million patients across 100 healthcare systems throughout the United States. The platform adheres strictly to the Health Insurance Portability and Accountability Act (HIPAA) guidelines and maintains certification under ISO 27001:2013, thus ensuring rigorous standards for patient confidentiality and data security [24, 25].

### Patient selection

**Fig. 1:**
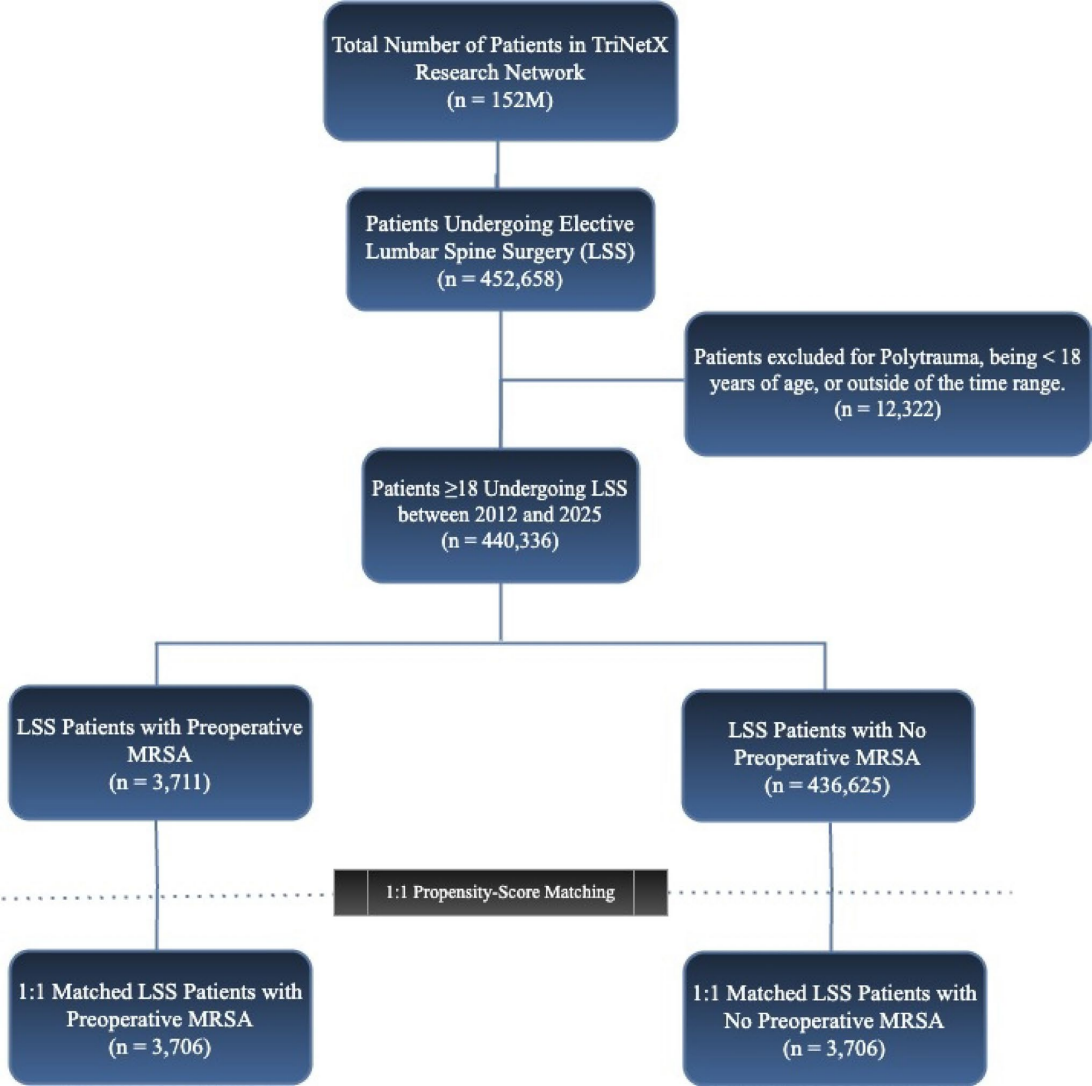
STROBE flowchart detailing patient selection

This retrospective cohort study included patients aged ≥ 18 years undergoing elective LSS procedures between 2012 and 2025. LSS procedures and preoperative diagnosis of MRSA were identified via validated CPT and ICD-10 codes provided in appendix A.1. Patients with documented preoperative MRSA colonization within the one-year period prior to their lumbar spine surgery (LSS) were included to ensure an accurate reflection of their MRSA status at the time of surgery. Patients were stratified into two groups: (1) MRSA-positive and (2) non-MRSA colonization (control group). To maintain the homogeneity of elective procedures and exclude trauma-related interventions, patients with a diagnosis of polytrauma (ICD-10: T07) recorded within one week preceding the LSS were excluded. This retrospective cohort study adhered to the Strengthening the reporting of observational studies in epidemiology (STROBE) guidelines for observational research, with the detailed patient selection flowchart presented in Fig. 1.

### Outcomes evaluated

30-Day postoperative outcomes analyzed encompassed all-cause mortality, stroke, cardiac arrest and ventricular fibrillation, heart failure, blood transfusions, anemia, acute kidney failure (AKF), deep vein thrombosis (DVT), pulmonary embolism (PE), myocardial infarction (MI), pneumonia, urinary tract infections (UTI), mortality, sepsis, superficial and deep SSIs, wound dehiscence.

### Statistical analysis

Risk ratios (RR) with corresponding 95% confidence intervals were calculated for all measured outcomes to compare event rates between the two cohorts, supplemented with absolute risk difference. Statistical significance was determined using Fisher’s exact test or Chi-square test for categorical variables and Student’s t-test for continuous variables. A p-value ≤ 0.05 was considered statistically significant. All statistical analyses were performed using R (version 4.3.2; R Foundation for Statistical Computing, Vienna, Austria), primarily utilizing the tidyverse and stats packages for exploratory analysis and hypothesis testing. Propensity score matching and additional cohort-level analyses were conducted within the TriNetX platform.

### Propensity-score matching

To minimize potential confounding, patients were matched in a 1:1 ratio using propensity score matching with a greedy nearest-neighbor algorithm without replacement. Matching was performed based on key covariates identified from preliminary regression analyses, including age at surgery, race, sex, obesity, diabetes mellitus, tobacco use, malnutrition, and chronic kidney disease. Following propensity matching, each cohort consisted of 3706 patients. Matching quality was verified by ensuring the standardized mean differences were below 0.1 for all matching variables, indicating successful balancing. Comprehensive comparison data of baseline characteristics before and after matching is presented in Appendix A.2.

## Results

### Patient demographics and characteristics analysis

A total of 440,336 patients who underwent LSS were identified from the TriNetX database within the specified study period. Among these patients, 3,711 (0.84%) tested positive for preoperative MRSA colonization and formed the MRSA-positive study group. The remaining 436,625 patients without MRSA colonization were designated as the control group (Table 1).

**Table 1:** Demographic and clinical characteristics of MRSA-positive and control patients

Variable	MRSA (+) (*n* = 3711) †	No MRSA (control) (*n* = 436,625) †	*p*	SMD
Age > 65	2074 (55.89%)	257,424 (58.96%)	0.092	0.062
Sex (proportion of women)	1690 (45.54%)	201,478 (46.14%)	0.462	0.012
Diabetes mellitus	1231 (33.2%)	72,550 (16.6%)	< 0.001	0.390
Tobacco use	302 (8.1%)	12,773 (2.9%)	< 0.001	0.230
Nicotine dependence	818 (22.0%)	37,144 (8.50%)	< 0.001	0.383
Obesity	1257 (33.9%)	74,836 (17.1%)	< 0.001	0.391
Malnutrition	435 (11.7%)	5415 (1.2%)	< 0.001	0.436
Chronic kidney disease (CKD)	572 (15.4%)	25,827 (5.9%)	< 0.001	0.311
Race/ethnicity	–	–	< 0.001*	–
White	2957 (79.7%)	313,867 (71.9%)	–	0.183
Hispanic or latino	147 (4.0%)	23,890 (5.5%)	–	0.071
American Indian or Alaska Native	22 (0.6%)	1421 (0.3%)	–	0.040
Black or African American	264 (7.1%)	34,757 (8.0%)	–	0.032
Asian	87 (2.3%)	28,884 (6.6%)	–	0.208
Native Hawaiian or other Pacific Islander	28 (0.8%)	1835 (0.4%)	–	0.044
Other race	74 (2.0%)	11,797 (2.7%)	–	0.047

*MRSA (+)* patient tested positive for preoperative colonization or infection with MRSA, *SMD* standardized mean difference, *MRSA* methicillin-resistant

† The values indicate the number of patients, with percentages shown in parentheses

* *p* < 0.05

Patients in the MRSA-positive group had a slightly lower proportion of individuals older than 65 years (55.89%) compared to the control group (58.96%), although this difference was not statistically significant (*p* = 0.092). The distribution of sex was comparable between groups, with women comprising 45.54% of the MRSA-positive group and 46.14% of the control group (*p* = 0.462) (Table 1).

Significant differences were noted in several other demographic characteristics. The MRSA-positive group had a substantially higher proportion of obesity (33.87%) compared to the control group (17.14%) (*p* < 0.001). Similarly, higher rates of tobacco use (22.04% vs. 8.51%, *p* < 0.001), diabetes mellitus (33.17% vs. 16.62%, *p* < 0.001), malnutrition (11.72% vs. 1.24%, *p* < 0.001), and chronic kidney disease (15.41% vs. 5.92%, *p* < 0.001) were observed in the MRSA-positive cohort (Table 1).

The overall distribution of race and ethnicity differed significantly between the MRSA-positive and control groups (*p* < 0.001, Chi-squared test, df = 6). Specifically, the MRSA-positive group had a higher proportion of White (79.7% vs. 71.9%) and American Indian or Alaska Native (0.6% vs. 0.3%) individuals, while the control group had higher proportions of Asian (6.6% vs. 2.3%) and Hispanic or Latino (5.5% vs. 4.0%) individuals. The proportions of Black or African American, Native Hawaiian or Other Pacific Islander, and Other Race were similar between groups (Table 1).

### Unmatched analysis of 30-day outcomes (Table 2 and Fig. 2)

The unmatched analysis demonstrated significantly higher risks of several postoperative complications in MRSA-colonized LSS patients compared to controls (Table 2). MRSA-positive patients exhibited notably increased rates of deep SSIs (0.90% vs. 0.20%, RR = 4.021, *p* < 0.001), superficial SSIs (0.70% vs. 0.20%, RR = 2.992, *p* < 0.001), PE (0.70% vs. 0.30%, RR = 2.240, *p* < 0.001), DVT (1.10% vs. 0.60%, RR = 1.764, *p* < 0.001), and wound dehiscence (2.00% vs. 0.90%, RR = 2.300, *p* < 0.001).

**Fig. 2:**
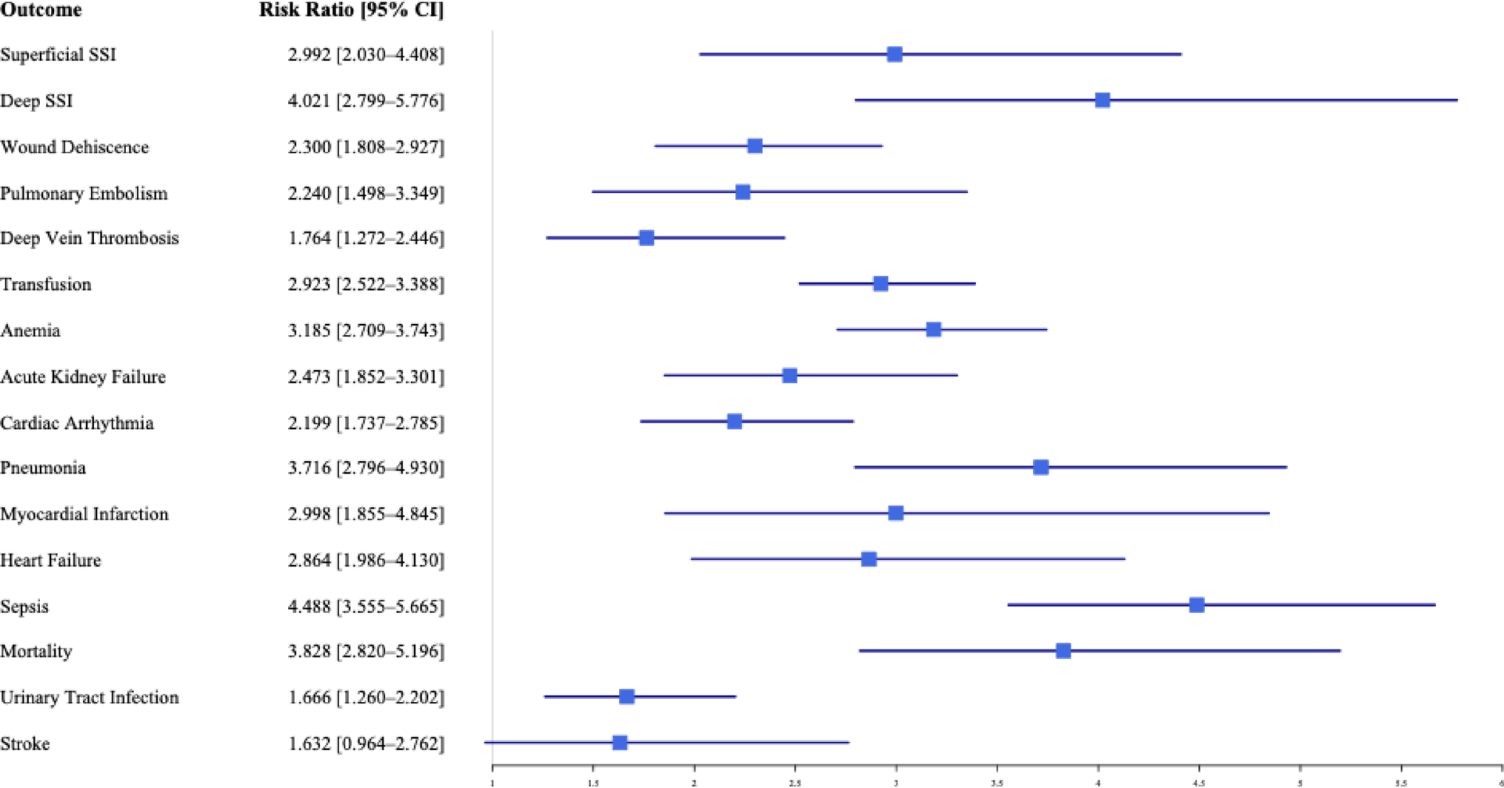
Forest plot of risk ratios for one-month outcomes-unmatched

**Table 2:** Unmatched analysis of postoperative complications in MRSA-positive vs. control patients

30 days-unmatched					
Measure	MRSA (+) †	Control group †	Risk ratio	95% CI	*p*-value
Stroke	14 (0.40%)	1085 (0.30%)	1.632	(0.964, 2.762)	0.07
Cardiac arrest and ventricular fibrillation	< 10 ‡	245 (0.10%)	–	–	–
Heart failure	29 (0.90%)	1351 (0.30%)	2.864	(1.986, 4.130)	**< 0.001**
Superficial surgical site infection	26 (0.70%)	1057 (0.20%)	2.992	(2.030, 4.408)	**< 0.001**
Deep surgical site infection	30 (0.90%)	914 (0.20%)	4.021	(2.799, 5.776)	**< 0.001**
Wound dehiscence	66 (2.00%)	3703 (0.90%)	2.300	(1.808, 2.927)	**< 0.001**
Pulmonary embolism	24 (0.70%)	1350 (0.30%)	2.240	(1.498, 3.349)	**< 0.001**
Deep vein thrombosis	36 (1.10%)	2625 (0.60%)	1.764	(1.272, 2.446)	**< 0.01**
Transfusion	170 (5.60%)	7895 (1.90%)	2.923	(2.522, 3.388)	**< 0.001**
Anemia	141 (6.00%)	7197 (1.90%)	3.185	(2.709, 3.743)	**< 0.001**
Acute kidney failure	46 (1.70%)	2736 (0.70%)	2.473	(1.852, 3.301)	**< 0.001**
Cardiac arrhythmia	68 (2.80%)	4530 (1.30%)	2.199	(1.737, 2.785)	**< 0.001**
Pneumonia	48 (1.60%)	1781 (0.40%)	3.716	(2.796, 4.938)	**< 0.001**
Myocardial infarction	17 (0.50%)	703 (0.20%)	2.998	(1.855, 4.845)	**< 0.001**
Mortality	42 (1.20%)	1307 (0.30%)	3.828	(2.820, 5.196)	**< 0.001**
Sepsis	71 (2.60%)	2431 (0.60%)	4.488	(3.555, 5.665)	**< 0.001**
Urinary tract infection	49 (1.80%)	4122 (1.10%)	1.666	(1.260, 2.202)	**< 0.001**

Significant values are bolded (*p* < 0.05)

† The values indicate the number of patients, with percentages shown in parentheses

‡ Patient counts of ≤ 10 are not reported on TriNetX

Additional significant differences included higher rates of anemia requiring transfusion (5.60% vs. 1.90%, RR = 2.923, *p* < 0.001), AKF (1.70% vs. 0.70%, RR = 2.473, *p* < 0.001), pneumonia (1.60% vs. 0.40%, RR = 3.716, *p* < 0.001), and sepsis (2.60% vs. 0.60%, RR = 4.488, *p* < 0.001). Elevated risks were also observed for cardiac complications such as MI (0.50% vs. 0.20%, RR = 2.998, *p* < 0.001), heart failure (0.90% vs. 0.30%, RR = 2.864, *p* < 0.001), and cardiac arrhythmia (2.80% vs. 1.30%, RR = 2.199, *p* < 0.001). MRSA-colonized patients also showed increased mortality rates (1.20% vs. 0.30%, RR = 3.828, *p* < 0.001).

The occurrence of stroke did not reach statistical significance (0.40% vs. 0.30%, *p* = 0.07), and data were insufficient to analyze cardiac arrest and ventricular fibrillation due to low event counts in the MRSA-positive group.

### Matched analysis of 30-day outcomes (Table 3 and Fig. 3)

In the matched analysis, MRSA-positive LSS patients continued to show significantly higher risks for several key postoperative complications compared to matched controls (Table 3). Notably, MRSA colonization was associated with increased rates of superficial SSIs (0.70% vs. 0.30%, RR = 2.291, *p* < 0.01), deep SSIs (0.80% vs. 0.30%, RR = 2.566, *p* < 0.01), wound dehiscence (2.00% vs. 1.20%, RR = 1.722, *p* < 0.01), and anemia requiring transfusion (5.40% vs. 2.30%, RR = 2.382, *p* < 0.001).

**Fig. 3:**
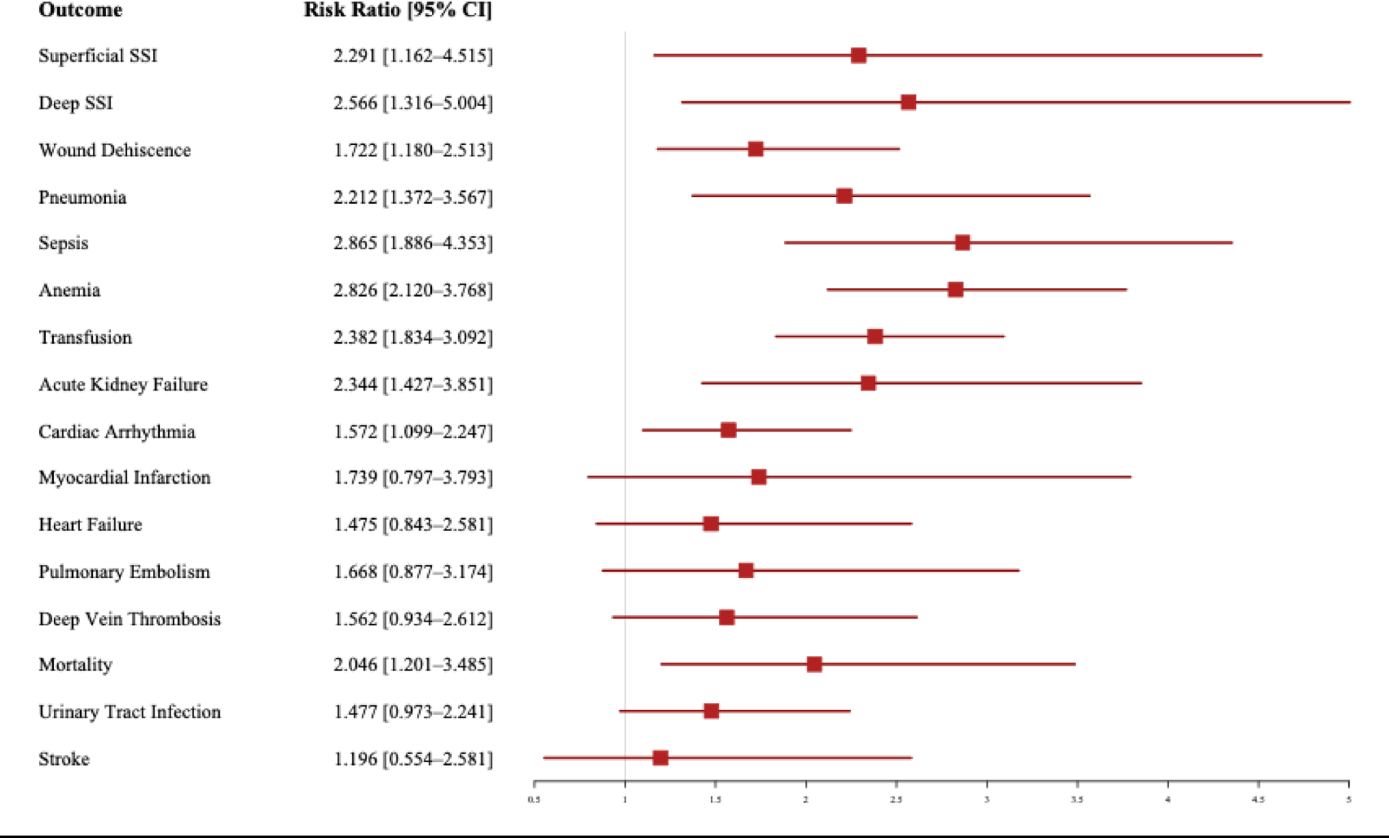
Forest plot of risk ratios for one-month outcomes- matched

**Table 3:** Matched analysis of postoperative complications in MRSA-positive vs. control patients

30 days-matched					
Measure	MRSA (+) †	Control group †	Risk ratio	95% CI	*p*-value
Stroke	14 (0.40%)	12 (0.40%)	1.196	(0.554, 2.581)	0.65
Cardiac arrest and ventricular fibrillation	< 10 ‡	< 10 ‡	–	–	–
Heart failure	29 (0.90%)	21 (0.60%)	1.475	(0.843, 2.581)	0.17
Superficial surgical site infection	27 (0.70%)	12 (0.30%)	2.291	(1.162, 4.515)	**< 0.01**
Deep surgical site infection	30 (0.80%)	12 (0.30%)	2.566	(1.316, 5.004)	**< 0.01**
Wound dehiscence	69 (2.00%)	43 (1.20%)	1.722	(1.180, 2.513)	**< 0.01**
Pulmonary embolism	24 (0.70%)	15 (0.40%)	1.668	(0.877, 3.174)	0.12
Deep vein thrombosis	36 (1.10%)	24 (0.70%)	1.562	(0.934, 2.612)	0.09
Transfusion	171 (5.40%)	80 (2.30%)	2.382	(1.834, 3.092)	**< 0.001**
Anemia	145 (6.00%)	65 (2.10%)	2.826	(2.120, 3.768)	**< 0.001**
Acute kidney failure	47 (1.60%)	23 (0.70%)	2.344	(1.427, 3.851)	**< 0.001**
Cardiac arrhythmia	71 (2.90%)	50 (1.80%)	1.572	(1.099, 2.247)	**< 0.01**
Pneumonia	50 (1.60%)	25 (0.70%)	2.212	(1.372, 3.567)	**< 0.001**
Myocardial infarction	17 (0.50%)	10 (0.30%)	1.739	(0.797, 3.793)	0.16
Mortality	41 (1.10%)	20 (0.50%)	2.046	(1.201, 3.485)	**< 0.01**
Sepsis	72 (2.50%)	31 (0.90%)	2.865	(1.886, 4.353)	**< 0.001**
Urinary tract infection	51 (1.80%)	38 (1.20%)	1.477	(0.973, 2.241)	0.07

Significant values are bolded (*p* < 0.05)

† The values indicate the number of patients, with percentages shown in parentheses

‡ Patient counts of ≤ 10 are not reported on TriNetX

Additional significant differences included AKF (1.60% vs. 0.70%, RR = 2.344, *p* < 0.001), pneumonia (1.60% vs. 0.70%, RR = 2.212, *p* < 0.001), cardiac arrhythmia (2.90% vs. 1.80%, RR = 1.572, *p* < 0.01), sepsis (2.50% vs. 0.90%, RR = 2.865, *p* < 0.001), and mortality (1.10% vs. 0.50%, RR = 2.046, *p* < 0.01).

Differences in stroke, heart failure, PE, DVT, MI, and UTI rates were not statistically significant. Data for cardiac arrest and ventricular fibrillation were insufficient due to low event counts in both groups.

## Discussion

This large-scale study of 440,336 patients undergoing elective LSS demonstrated that preoperative MRSA colonization was associated with significantly elevated risks of all-cause mortality, wound-related complications (including superficial and deep SSIs), systemic infectious sequelae, hematologic morbidity, and acute renal injury following LSS. These findings underscore that preoperative identification of MRSA colonization may enable orthopaedic surgeons to proactively optimize perioperative management protocols and deliver targeted counseling to LSS patients regarding their heightened complication risks [26, 38].

### Wound-related complications

Wound-related morbidity demonstrated significantly elevated risks in MRSA-colonized patients compared to matched controls. Superficial SSIs occurred at 2.29 times the rate observed in non-colonized controls (0.70% vs. 0.30%; RR = 2.291, *p* < 0.01), while deep SSIs manifested at 2.57 times the control rate (0.80% vs. 0.30%; RR = 2.566, *p* < 0.01). Notably, wound dehiscence affected 2.00% of MRSA carriers versus 1.20% of controls (RR = 1.722, *p* < 0.01), constituting a 72% relative risk elevation.

The pathophysiological basis for these outcomes may be centered around MRSA’s distinct virulence profile. Prior evidence indicates MRSA rapidly forms antibiotic-resistant biofilms on orthopaedic hardware, compromising host defenses and complicating eradication [26]. Furthermore, toxin production, particularly α-hemolysin and Panton-Valentine leukocidin (PVL), induces leukocyte destruction and tissue necrosis, potentially impairing wound healing [26, 27]. Concurrent immune evasion mechanisms, including suppression of opsonization and phagocytosis through virulence factors, may also facilitate persistent surgical site colonization [28], and collectively, these processes promote recalcitrant infections and delayed tissue recovery.

Contemporary spine literature substantiates this biological framework as Ning et al.‘s meta-analysis of 12,214 patients demonstrated a 6.2-fold increased risk of MRSA-specific SSIs (95% CI 3.4–11.3) among nasally colonized patients [29], while Abdul-Jabbar et al. identified MRSA as the predominant pathogen in 45% of all instrumented spine SSIs in their study [30]. This association also extends beyond spinal procedures, as Areti et al. documented a 2.13-fold elevated SSI risk (OR 2.13, *p* = 0.008) in colonized total knee arthroplasty patients [11].

Clinically, these findings may support the use of protocolized preoperative MRSA screening via PCR-based assays of nares and perineal swabs, aligning with the Infectious Diseases Society of America’s (IDSA) 2022 guideline recommendation for targeted decolonization using intranasal mupirocin and chlorhexidine gluconate bathing in orthopaedic surgery patients [1]. Moreover, evidence-based decolonization regimens have also been found to be associated with significant reductions in SSIs, consistent with systematic reviews reporting relative risk reductions of 13–200% for overall SSIs and 40–200% for *S. aureus* SSIs in orthopaedic cohorts [31–33].

### Systemic infectious complications

Beyond localized wound morbidity, MRSA colonization was also associated with increased risk of systemic infectious complications in our study. Colonized patients exhibited a 2.21-fold higher incidence of postoperative pneumonia (1.60% vs. 0.70%; RR = 2.21, *p* < 0.001) and a 2.87-fold greater risk of sepsis (2.50% vs. 0.90%; RR = 2.87, *p* < 0.001) compared to non-colonized controls.

The progression from colonization to systemic dissemination may involve distinct pathophysiological mechanisms as hematogenous spread is known to be facilitated by MRSA surface proteins that mediate endothelial binding and transmigration across vascular barriers [34]. Pulmonary infiltration may also trigger toxin-mediated immunopathology, with virulence factors inducing neutrophil lysis and alveolar damage characteristic of necrotizing pneumonia [35]. Furthermore, MRSA is also known to provoke dysregulated cytokine cascades, notably elevated interleukin-6 and tumor necrosis factor-α (TNF-α) production exceeding responses to other pathogens, and this hyperinflammatory state could promoted disseminated intravascular coagulation and potentiate sepsis/ septic shock [36, 37].

Orthopaedic literature across spinal and non-spinal contexts corroborates these findings as Saad Berreta et al. documented 49% greater pneumonia risk in spinal MRSA infections versus MSSA controls (OR = 1.49, *p* = 0.002), implicating hematogenous seeding from surgical sites [26]. Hardtstock et al.‘s registry analysis of 23,485 procedures further demonstrated that MRSA-infected patients faced substantially elevated 1-year mortality (22.4% vs. 5.3%, *p* < 0.001), with systemic infections identified as primary contributors to this excess mortality [38].

### Hematologic complications

Concerning hematologic outcomes, MRSA-colonized patients demonstrated significantly elevated incidence rates of anemia and transfusion relative to the control cohort. Postoperative anemia occurred in 6.00% versus 2.10% of controls (RR = 2.83, *p* < 0.001), while transfusion requirements manifested in 5.40% versus 2.30% (RR = 2.38, *p* < 0.001), representing 2.83- and 2.38-fold risk elevations, respectively.

Mechanistically, these findings are not unexpected given that the chronic inflammatory states induced by MRSA colonization may disrupt iron homeostasis through hepcidin-mediated pathways, suppressing erythropoiesis [39, 40]. Concurrently, MRSA colonization also promotes localized microvascular thrombosis via coagulase production and platelet activation, potentially causing tissue hypoxia and potentiating surgical blood loss [41, 42]. This dual pathophysiology may underlie the observed increase in transfusion requirements in MRSA colonized patients.

While spine-specific literature on MRSA hematologic effects remains limited, broader orthopaedic evidence suggests similar significant associations. Dreyfus et al.‘s analysis of 419,200 procedures identified preoperative anemia as an independent predictor of SSIs (OR = 3.1, 95% CI 2.4-4.0), establishing a clinically relevant bidirectional association that may exacerbate hematologic complications in MRSA carriers [42].

### Renal complications

MRSA-colonized patients in our study also exhibited significantly elevated renal morbidity relative to the control cohort. AKF developed in 1.60% versus 0.70% of controls (RR = 2.34, *p* < 0.001), representing a 2.34-fold risk elevation that persisted after comprehensive confounder adjustment.

The observed nephrotoxicity may arise from established synergistic mechanisms as direct cytotoxic effects of MRSA toxins, including pore-forming proteins and superantigens, are documented to induce tubular and glomerular cell death via mitochondrial dysfunction and disruption of cellular homeostasis, as demonstrated in models of staphylococcal renal injury [43, 44]. Concurrently, sepsis-related hypotension impairs renal perfusion, reducing glomerular filtration rate and oxygen delivery [45]. This macrocirculatory compromise is exacerbated by microvascular dysfunction characterized by endothelial injury, capillary leakage, and microthrombi formation, culminating in heterogeneous tissue hypoxia and progressive functional decline in renal structures [45–47].

Our findings align with broader spine-specific evidence recent investigations have also identified AKF to be significantly more prevalent in spinal MRSA infections compared to controls (OR = 1.35, *p* = 0.001) [26].

### Mortality

Regarding mortality, MRSA-colonized patients exhibited a 2.05-fold increase in 30-day mortality (1.10% vs. 0.50%; RR = 2.046, *p* < 0.01). Notably, this excess mortality may stem from the compounded burden of fulminant septic shock, particularly given the 2.87-fold sepsis risk observed in our cohort, combined with multisystem organ failure arising from the increased risk for synergistic complications discussed earlier. Though studies specific to MRSA-colonized LSS patients evaluating mortality remain limited, our findings closely align with Hardtstock et al.‘s registry analysis, demonstrating 1.72-fold higher 1-year mortality in MRSA-positive patients undergoing various orthopaedic procedures (adjusted HR = 1.72, 95% CI 1.32–2.24) [38].

Given the substantial morbidity and mortality associated with MRSA colonization demonstrated in this study, evaluating the economic implications of risk mitigation strategies is critical. In addition to its clinical relevance, routine preoperative MRSA screening may be a cost-effective strategy in elective spine surgery. PCR-based nasal swab screening is low-cost and can be integrated into standard preoperative workflows with minimal burden [48]. Given the significantly elevated risks of postoperative wound infection, sepsis, and transfusion in MRSA-colonized patients demonstrated in this study, early identification may reduce downstream costs associated with readmissions, reoperations, and prolonged hospitalizations. Prior economic modeling in orthopaedic surgery has shown that even selective decolonization protocols can yield substantial net savings when factoring in the costs of treating deep SSIs and MRSA-related complications [32, 33]. When coupled with effective decolonization regimens, targeted MRSA screening has the potential to improve both patient outcomes and healthcare resource utilization in elective lumbar spine surgery.

### Limitations

Several inherent limitations warrant consideration in this retrospective analysis. Our utilization of the TriNetX database may have precluded a granular assessment of institutional MRSA screening methodologies, resulting in misclassification of unscreened patients as MRSA-negative, and potential inaccuracies in ICD coding related to clerical errors or diagnostic inconsistency may have affected complication ascertainment reliability. Additionally, while propensity score matching adjusted for key comorbidities, unmeasured confounders such as patient frailty, functional status, and perioperative variables such as operative duration, blood loss, etc. may have remained unaccounted for due to database constraints. Moreover, heterogeneous MRSA screening practices across U.S. healthcare institutions could also contribute to detection bias, as hospitals employ individualized risk-based protocols rather than standardized national guidelines. In line with this variability, we were also unable to determine whether MRSA-colonized patients in our cohort underwent preoperative decolonization therapy. TriNetX does not provide standardized codes or data elements specific to treatments such as intranasal mupirocin or chlorhexidine bathing. As such, some patients classified as MRSA-positive may have completed decolonization protocols prior to surgery, potentially affecting their postoperative risk profile. This inability to account for decolonization status represents a limitation in accurately stratifying patients by true colonization risk. Lastly, our investigation did not incorporate validated preoperative health assessment measures, such as the American Society of Anesthesiologists (ASA) classification, potentially limiting a more detailed understanding of the baseline health status among MRSA-colonized patients. Future prospective studies should quantify how protocolized MRSA decolonization modifies these excess risks, potentially transforming risk-stratified perioperative management for spinal surgery patients.

## Conclusions

Our study demonstrated that preoperative MRSA colonization was associated with significantly increased risk of adverse postoperative complications following elective LSS, specifically wound-related complications such as superficial and deep SSIs and wound dehiscence, systemic infections including pneumonia and sepsis, hematologic events like anemia and transfusions, AKF, as well as all-cause mortality. The patient demographics and characteristics analysis also revealed 5 major risk factors, including the presence of obesity, tobacco use, diabetes mellitus, malnutrition, and chronic kidney disease, as independent predictors of MRSA colonization. Clinically, preoperative recognition of MRSA colonization could prompt implementation of multimodal decolonization protocols and targeted counseling regarding heightened complication risks. This risk-stratified approach may optimize perioperative management and improve outcomes in high-risk LSS patients.

## Supplementary Information

Below is the link to the electronic supplementary material.

Supplementary Material 1

## Data Availability

The datasets generated and analyzed during the current study are available from the corresponding author (Sri Tummala) upon reasonable request.
