# Supplementary material for: Evaluating the Risk of Postoperative Infection and Complications in Lumbar Spine Surgery Patients with Preoperative Methicillin-resistant Staphylococcus aureus (MRSA) Colonization
Source: Arch Orthop Trauma Surg. 2025 Aug 19;145(1):409. doi: 10.1007/s00402-025-06036-y (PMC12364758; doi:10.1007/s00402-025-06036-y)
Supplement: Supplementary file 1 — Supplementary Material 1 [file 402_2025_6036_MOESM1_ESM.docx]

**Appendix A:**

Appendix A.1.

**CPT and ICD-10 Codes for LSS Procedures:**

- Arthrodesis: CPT: 22612, 22533, 22630, 22633 ICD-10-PCS: 0SG0, 0SG1, 0SG3; Facetectomy ICD-10-PCS: 0SB40ZZ, 0SB43ZZ, 0SB44ZZ; Foraminotomy ICD-10-PCS: 0SB20ZZ, 0SB23ZZ, 0SB24ZZ; Laminectomy CPT: 63005, 63017, 63047 ICD-10-PCS: 0SB00ZZ, 0SB03ZZ, 0SB04ZZ; Laminotomy CPT: 63030, 63042 ICD-10-PCS: 0SB30ZZ, 0SB33ZZ, 0SB34ZZ; Disc/Joint Excision ICD-10-PCS: 1036727

**ICD-10 Codes for MRSA Diagnosis:**

- Z22.322, A49.02, B95.62

Appendix A.2.

**Patient demographics and characteristics before and after propensity-matching**

| Variable | *MRSA (n = 3,711) †* | *No MRSA (n = 436,625) †* | *p* | *SMD* | *Variable* | *MRSA (n = 3,706) †* | *No MRSA (n = 3,706) †* | *p* | *SMD* |
| --- | --- | --- | --- | --- | --- | --- | --- | --- | --- |
| Age at Index ‡ | *57.9 ± 14.7* | *58.2 ± 16.1* | *0.237* | *0.020* | *Age at index ‡* | *57.8 ± 14.7* | *58.2 ± 14.8* | *0.322* | *0.023* |
| Female | *1690 (45.5%)* | *201478 (46.1%)* | *0.462* | *0.012* | *Female* | *1686 (45.5%)* | *1693 (45.7%)* | *0.870* | *0.004* |
| Diabetes mellitus | *1231 (33.2%)* | *72550 (16.6%)* | *<0.001* | *0.390* | *Diabetes mellitus* | *1226 (33.1%)* | *1236 (33.4%)* | *0.805* | *0.006* |
| Tobacco Use | *302 (8.1%)* | *12773 (2.9%)* | *<0.001* | *0.230* | *Tobacco Use* | *298 (8.0%)* | *281 (7.6%)* | *0.462* | *0.017* |
| Nicotine dependence | *818 (22.0%)* | *37,144 (8.50%)* | *<0.001* | *0.383* | *Nicotine dependence* | *813 (21.9%)* | *820 (22.1%)* | *0.844* | *0.005* |
| Obesity | *1257 (33.9%)* | *74836 (17.1%)* | *<0.001* | *0.391* | *Obesity* | *1252 (33.8%)* | *1278 (34.5%)* | *0.524* | *0.015* |
| Malnutrition | *435 (11.7%)* | *5415 (1.2%)* | *<0.001* | *0.436* | *Malnutrition* | *430 (11.6%)* | *410 (11.1%)* | *0.464* | *0.017* |
| Chronic Kidney Disease (CKD) | *572 (15.4%)* | *25827 (5.9%)* | *<0.001* | *0.311* | *Chronic Kidney Disease (CKD)* | *567 (15.3%)* | *559 (15.1%)* | *0.796* | *0.006* |
| Race/Ethnicity | *-* | *-* | ***<0.001**** | *-* | *Race/Ethnicity* | *-* | *-* | ***0.631**** | *-* |
| White | *2957 (79.7%)* | *313867 (71.9%)* | *-* | *0.183* | *White* | *2952 (79.7%)* | *2988 (80.6%)* | *-* | *0.024* |
| Hispanic or Latino | *147 (4.0%)* | *23890 (5.5%)* | *-* | *0.071* | *Hispanic or Latino* | *147 (4.0%)* | *117 (3.2%)* | *-* | *0.044* |
| American Indian or Alaska Native | *22 (0.6%)* | *1421 (0.3%)* | *-* | *0.040* | *American Indian or Alaska Native* | *22 (0.6%)* | *23 (0.6%)* | *-* | *0.003* |
| Black or African American | *264 (7.1%)* | *34757 (8.0%)* | *-* | *0.032* | *Black or African American* | *264 (7.1%)* | *258 (7.0%)* | *-* | *0.006* |
| Asian | *87 (2.3%)* | *28884 (6.6%)* | *-* | *0.208* | *Asian* | *87 (2.3%)* | *79 (2.1%)* | *-* | *0.015* |
| Native Hawaiian or Other Pacific Islander | *28 (0.8%)* | *1835 (0.4%)* | *-* | *0.044* | *Native Hawaiian or Other Pacific Islander* | *28 (0.8%)* | *20 (0.5%)* | *-* | *0.027* |
| Other Race | *74 (2.0%)* | *11797 (2.7%)* | *-* | *0.047* | *Other Race* | *74 (2.0%)* | *61 (1.6%)* | *-* | *0.026* |
| † The values indicate the number of patients, with percentages shown in parentheses. ‡ The values indicate the mean age ± standard deviation. SMD = Standardized Mean Difference; MRSA = Methicillin-Resistant Staphylococcus Aureus. * Race/ethnicity p-value reflects a global Chi-squared test with 6 degrees of freedom across mutually exclusive categories. | | | | | | | | | |
